# Epidemiology of Visceral Leishmaniasis in a Reemerging Focus of Intense Transmission in Minas Gerais State, Brazil

**DOI:** 10.1155/2013/405083

**Published:** 2013-08-13

**Authors:** Ricardo Andrade Barata, Jennifer Cunha Peixoto, Aline Tanure, Marcela Esteves Gomes, Estefânia Conceição Apolinário, Emerson Cotta Bodevan, Holbiano Saraiva de Araújo, Edelberto Santos Dias, Aimara da Costa Pinheiro

**Affiliations:** ^1^Universidade Federal dos Vales dos Jequitinhonha e Mucuri, Diamantina, MG, Brazil; ^2^HSA Gestão e Projeto Ambiental Ltda, Belo Horizonte, MG, Brazil; ^3^Centro de Pesquisas René Rachou/FIOCRUZ, Belo Horizonte, MG, Brazil; ^4^Secretaria Municipal de Saúde, Governador Valadares, MG, Brazil

## Abstract

This study was developed in the urban area of Governador Valadares, a reemerging focus of intense transmission of visceral leishmaniasis (VL) in Brazil, presenting 86 human cases of VL from 2008 to 2011. The disease prevailed in males (73.2%) with most patients between 0 and 9 years (44.1%) and a lethality rate of 16.2%. A canine survey was carried out on 16,529 domestic dogs in 35 districts in the area and it showed that 30.2% of them (4,992 dogs) were positive for VL by serum assays. Prevalence ratios for canine VL varied between 13.6% and 53.4%. The clinical exam of 343 seropositive dogs showed that 49.9% of them were considered symptomatic, with larger prevalence of canine VL being in short-furred animals (90%). The entomological survey was performed in eight districts, where 2,539 phlebotomines were captured, preferentially in the peridomicile (84.5%). *Lutzomyia longipalpis* was the predominant species (90%) suggesting its participation in the VL transmission in the area. The correlation between canine prevalence and *L. longipalpis* density was evaluated.

## 1. Introduction

In Brazil, visceral leishmaniasis (VL) or kala-azar is one of the biggest concerns of public health due to its high morbid-mortality in nontreated cases [1]. The etiological agent *Leishmania infantum chagasi* is transmitted mainly through the bite of *Lutzomyia longipalpis* (Diptera, Psychodidae, Phlebotominae), which is infected when ingesting intracellular parasites from the peripheral blood of a dog (*Canis familiaris*), considered not only the main domestic reservoir but also an essential link for the maintenance of the VL epidemiological chain in urban areas [2–4]. 

In the last decades, the environmental modifications caused by man, the deforestation, the disordered city growth, the concomitant presence of *L. longipalpis,* and domestic animals, not to mention the precarious habitation conditions of the population, all contributed to the urbanization and geographical expansion of VL in Brazil and the emergence of new focuses or reactivation of old ones [5]. 

In Governador Valadares, located in Rio Doce Valey, MG, cases of VL were registered in the 60s [6]. When the visceral leishmaniasis control program was adopted, the municipality was considered as belonging to a “controlled endemic” area. However, in the beginning of the 90s, the program was interrupted and epidemiological surveillance has not been carried out regularly in the region since then, without notification of human cases of VL up to 2007. 

Since 2008, human cases of VL began to be registered in the municipality of Governador Valadares. Given this situation, the present study undertakes an epidemiological investigation taking into account human and canine cases and also the phlebotomine fauna, in a reemergent focus of intense VL transmission in Brazil. 

## 2. Materials and Methods

### 2.1. Study Area

The municipality of Governador Valadares (18°51′12′′ S–41°56′42′′ W) is located in the eastern region of Minas Gerais state, covering part of the Rio Doce basin, in the southeastern area of Brazil. The city has 263,689 inhabitants distributed in 150 districts [7]. The region is subjected to a hot and humid climate in which temperatures vary little throughout the year (annual temperature average of 25.6°C). Its topography is characterized by a hilly relief. The vegetation is between the ecosystems of seasonal semidecidual forest and savannah, but due to logging in the past, some native species were replaced by pastures, with some representatives in areas of environmental protection in the municipal surrounding areas. 

### 2.2. Human Cases of VL

Data concerning the number of human cases of VL in the urban area of Governador Valadares from 2008 to 2011 was obtained from the Epidemiology Management/DVS/SMS, that considered the municipality as an area of intense VL transmission (average of cases in the last 5 years ≥ 4.4 cases) in the state of Minas Gerais [5]. 

### 2.3. Canine VL Survey

The canine VL (CVL) survey was carried out from 2008 to 2011 in all dogs domiciled in neighborhoods in the urban area with records of human cases in Governador Valadares. Blood samples were obtained annually in all the districts through the cervical or jugular vein puncture for serum production. A first screening for the presence of anti-*Leishmania* immunoglobulin in canine serum was performed by ELISA (Bio-Manguinhos/Fiocruz, RJ, Brazil) [8]. The diagnosis confirmation was accomplished through the indirect immunofluorescence assay (IFAT) [9], in agreement with the procedure adopted by the Brazilian Ministry of Health [5]. Among the positive animals for IFAT, a random sample of 343 dogs was analyzed according to the clinical characteristics of the animal. They were organized into asymptomatic or symptomatic groups, according to the absence or presence of at least one sign of VL infection (i.e., cutaneous lesions, onychogryphosis, keratoconjunctivitis, weight loss, emaciation, and rigidity of subsequent limbs) [10]. In addition, they were classified in short-furred (i.e., Basset, Beagle, Boxer, Brazilian Fila, Doberman, Pinscher, Pit-bull, Rottweiler, Shar pei, Weimaraner, and Mongrel) and long-furred dogs (i.e., Cocker, Siberian husky, Labrador, Lhasa-apso, German Shepherd, and Poodle).

### 2.4. Entomological Captures

Phlebotomine captures were performed in 8 neighborhoods in the municipality of Governador Valadares (Altinópolis, Mãe de Deus, Nossa Senhora das Graças, Santa Helena, Ilha de Araújos, Santos Dumont, São Raimundo, and Turmalina). The neighborhoods were chosen taking into account the prevalence rates canine (high and low). Thirty-two HP light traps [11] were distributed in the intra- and peridomicile, exposed in two residences of each neighborhood. The residences were chosen according to favorable ecological conditions for their development, such as the presence of trees, domestic animals, and organic matter. Sand flies were captured with traps assembled at 4:00 pm and removed the following day at 8:00 am, for 3 consecutive nights each month, between May 2011 and January 2012. The specimens captured were conditioned into hemolysis tubes containing alcohol (70%) and in accordance with Young and Duncan [12].

### 2.5. Statistical Analysis

The Shapiro-Wilk test was used to evaluate the normality of data distribution (Software: R v. 2.14.1). The Spearman correlation between the prevalence of canine infection and the density of *L*.* longipalpis* was calculated and its statistical significance assessed (*P*  value ≤ 0.05).

### 2.6. Ethical Aspects

This study was conducted in accordance with the ethical principles of animal experimentation adopted by the Brazilian College of Animal Experimentation (COBEA) and it was approved by the Ethical Committees on the Use of Animals of Universidade Federal dos Vales do Jequitinhonha e Mucuri (CEUA/UFVJM 010/10, Diamantina, Brazil).

## 3. Results

The municipality of Governador Valadares presented 86 autochthonous cases of visceral leishmaniasis from 2008 to 2011, with 14 cases in 2008, 27 in 2009, 24 in 2010, and 21 in 2011 (data not showed). The disease prevailed in males (73.2%), in children aged 0–9 years (44.1%) with a lethality rate of 16.2% (Table 1). 

**Figure 1:**
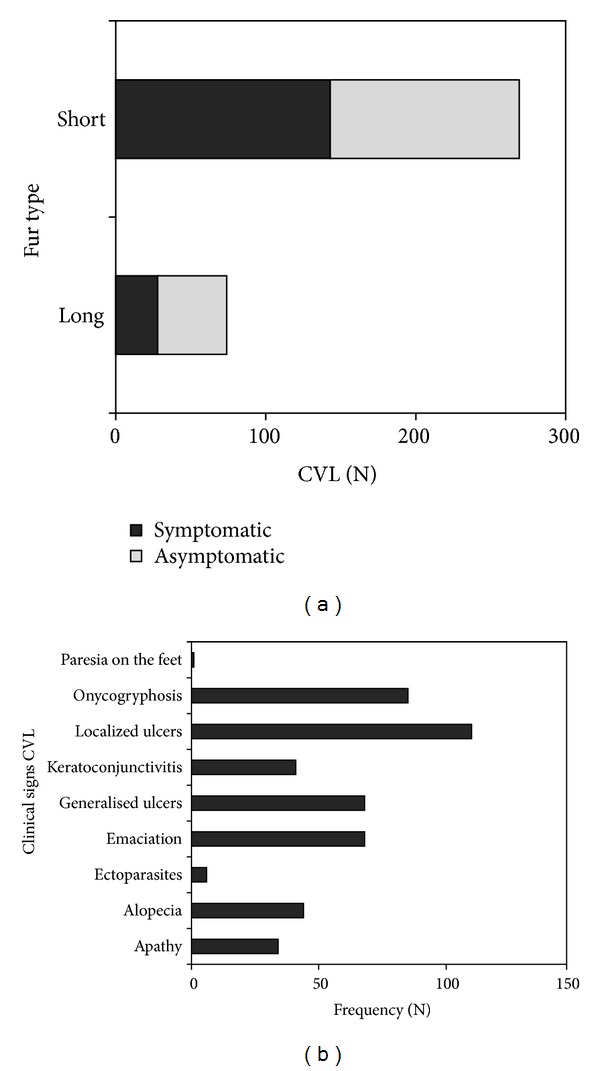
Frequencies of canine visceral leishmaniasis by fur type and clinical status (a) and the recorded clinical signs of CVL (b) in urban dogs of Governador Valadares, MG, Brazil.

The canine survey carried out in 35 districts of Governador Valadares from 2008 to 2011 resulted in 4,992 (30.2%) VL-seropositive dogs among 16,529 tested (Table 2). Canine VL was widespread throughout the urban area, with prevalence rates varying from 13.6% (São Paulo district) to 53.4% (Carapina district) (Table 2). Among the 343 seropositive dog samples, 49.9% were considered symptomatic, with a larger prevalence of CVL in short-furred dogs (90%) (Figure 1(a)). The most frequent clinical signs in symptomatic animals were located ulcers and onycohgryphosis, as evidenced in Figure 1(b). 

The phlebotomine fauna in Governador Valadares urban area consisted of 4 species: *Lutzomyia cortelezzii* (Brèthes, 1923), *Lutzomyia intermedia* (Lutz and Neiva, 1912), *Lutzomyia longipalpis* (Lutz and Neiva, 1912), and *Lutzomyia whitmani* (Antunes and Coutinho, 1939), totaling 2,539 specimens, of which 2,105 were males (83%) and 434 females (17%). The peridomicile presented the largest percentage of the captured specimens (84.5%). The predominant species was *L. longipalpis* (90%) (Table 3). The phlebotomine distribution according to the neighborhoods and months of collection can be visualized in Table 4. 

**Figure 2:**
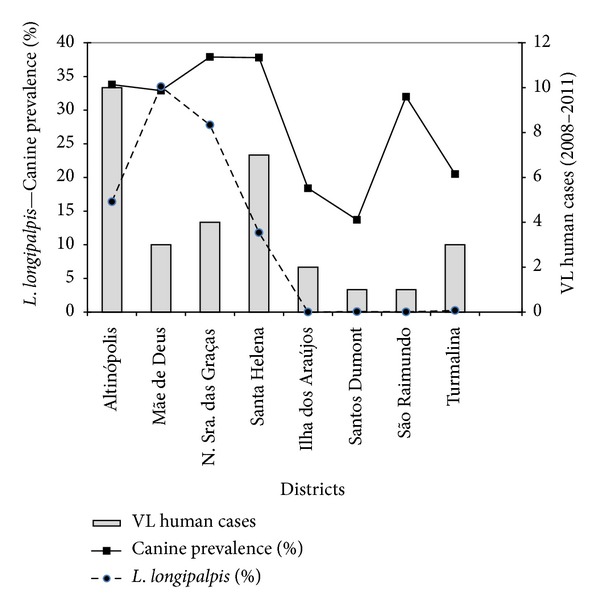
Distribution of *Lutzomyia longipalpis* population, prevalence of canine infection, and human cases VL per district of Governador Valadares, MG, Brazil.

The correlation between the density of the *L. longipalpis* captured and canine prevalence is represented in Figure 2. There was no statistically significant association between the variables number of sandflies and prevalence of canine infection (*P* = 0.057).

## 4. Discussion

In Brazil, VL is considered a neglected disease that prevails in places where underprivileged social conditions predominate. It contributes to the maintenance of the inequality picture that we have nowadays, as it represents a strong barrier to the social and economical development of the country [5]. In the last decades, the urbanization phenomenon has been pointed out as the responsible for the appearance of new focuses and reemergence of old ones in urban areas of small and medium-sized cities [13–15]. 

Governador Valadares, located in the Southeastern area of Brazil, is an example of a reemerging focus of VL as a result of urbanization. The VL cases notified occurred where housing conditions, basic sanitation, and garbage collection were poor. The residents have low socioeconomic status, living together with domestic animals and accumulated organic matter (data not shown). The same conditions were evidenced in other places where VL transmission is endemic [16, 17]. 

Analyzing the human cases according to the age group it was noticed that VL was more frequent in children under 10 years old (44.1%) (Table 1). The same findings were reported by several authors [18–20]. The lethality rate was 16.2%. Nascimento et al. [14] and Queiroz et al. [21] verified a lethality rate of 11.5% and 10.2%, respectively. The greatest prevalence of VL in children (Table 1) can explain the high lethality rate found, as they are yet incompletely developed immune system.

The prevalence of canine-VL in Brazil has been demonstrated to be between 1.9 and 35% in endemic areas [22–26]. In a previous study undertaken in Governador Valadares, Malaquias et al. [27] evidenced 13.7% of positivity in dogs in urban areas. In the present study, the average prevalence was 30.2%, but in some neighborhood it reached 53.4%. The high prevalence of canine infection has been given as one of the risk factors for VL occurrence [28, 29]. 

In this context, symptomatic or asymptomatic seropositive dogs play an important role in the maintenance of the infection [30]. Signs of VL were detected in 171 (49.9%) of the 343 dogs sampled. The pattern of clinical signs observed for the disease most commonly included weight loss, apathy, and emaciation, similar to what was observed by Silva et al. [15]. The most worrying fact is the percentage of dogs with nonapparent infection (50.1%), as they show a high degree of cutaneous parasitism, being capable of living together with the parasite for long periods [31, 32]. 

In addition, short-furred dogs represented the largest percentage of seropositive animals (90%), suggesting that short fur determines larger chance by a greater propensity to contract the infection in the population investigated because it enables the phlebotomine access to the place of the bite [33, 34]. These results are reinforced by França-Silva et al. [4] who observed a larger prevalence of CVL in short-furred dogs.

In Minas Gerais, several authors have showed the abundance of *L. longipalpis* in urban areas where VL is endemic [16, 35, 36]. The same pattern has been observed in other Brazilian areas [37, 38] where the species clearly participates in the transmission of *L. infantum chagasi*. However, in previous research in the same area as the present study, although the authors found *L. longipalpis*, it was not the most abundant species. This fact may be because the area sampled was a transitional one between wild and urban environments. Thus the predominance of this sand fly in an urban area with high frequencies in residences and surroundings suggests its participation in the transmission of *L. infantum chagasi* among dogs and humans in the city of Governador Valadares [39]. 

The presence of a great number of seropositive dogs and the high density of *L. longipalpis* have been affirmed to be the main risk factors for the occurrence of VL in urban areas [40, 41]. In the present study, the correlation between these two variables presented no statistical significance. However, if we analyze Figure 2, we notice that there is a tendency for an increase in canine prevalence in neighborhoods where the density of *L. longipalpis* is higher, such as Altinópolis, Nossa Senhora das Graças, Mãe de Deus, and Santa Helena. 

Thus, these factors seem to be decisive for the occurrence of VL in the city of Governador Valadares, reinforcing the need for rigid controlling actions through the euthanasia of seropositive dogs, the use of residual insecticide, and environmental management in residences, as well as rigorous epidemiological surveillance. 

## Figures and Tables

**Table 1:** Distribution of human cases of VL according to age, sex, and lethality in Governador Valadares from 2008 to 2011.

Age group (years)	VL human cases		Total	%	Lethality (*N*)
	Female	Male			
0–9	15	23	38	44.1	5
10–19	3	4	7	8.2	1
20–29	1	3	4	4.7	1
30–39	1	7	8	9.3	1
40–49	1	16	17	19.8	2
50–59	0	6	6	6.9	1
60–69	2	3	5	5.8	2
>70	0	1	1	1.2	1

Total	23	63	86	100	14

**Table 2:** Median prevalence of canine visceral leishmaniasis infection and number of human cases, by district, in Governador Valadares from 2008 to 2011.

Districts	Number of dogs examined	IFAT-positive dogs	Prevalence (%)	VL human cases
Altinópolis	2,237	756	33.8	10
Atalaia	47	18	38.3	1
Carapina	189	101	53.4	2
Centro	605	121	20.0	6
Esperança	274	104	37.9	2
Esplanada	166	85	51.2	1
Fraternidade	262	63	24.0	1
Grã-Duquesa	891	332	37.2	2
Ilha dos Araújos	690	127	18.4	2
JK	259	58	22.4	1
Jardim do Trevo	683	185	27.1	3
Lourdes	824	327	39.7	6
Maria Eugênia	205	69	33.6	1
Mãe de Deus	401	132	32.9	3
Monte Carmelo	NU	NU	—	1
N. Sra. das Graças	725	275	37.9	4
Palmeiras	764	238	31.1	7
Planalto	354	124	35.0	2
Santa Efigênia	195	54	27.7	1
Santa Helena	1,536	581	37.8	7
Santa Terezinha	342	90	26.3	1
Santo Antônio	519	148	28.5	3
Santos Dumont	256	35	13.7	1
São Cristóvão	268	64	23.9	2
São José	165	24	14.5	1
São Paulo	785	107	13.6	1
São Raimundo	25	8	32.0	1
Turmalina	946	194	20.5	3
Vila Bretas	528	116	21.9	4
Vila Império	226	46	20.3	1
Vila Isa	230	68	29.5	1
Vila Mariana	460	218	47.4	1
Vila dos Montes	19	6	31.6	1
Vila Ozanã	161	48	29.8	1
Vila Rica	292	70	24.0	1

Total	16,529	4,992	30.2	86

NU: not undertaken.

**Table 3:** Phlebotomine sandflies captured with HP trap in Governador Valadares by species, sex, and environment (from May 2011 to January 2012).

Species	Environment				Total	%
	Inside		Outside	
	♀	♂	♀	♂
*Lutzomyia cortelezzii *	26	13	79	63	181	7.1
*L. intermedia *	3	2	3	10	18	0.7
*L. longipalpis *	80	250	221	1,733	2,284	90.0
*L. whitmani *	1	2	0	1	4	0.1
*Lutzomyia *spp.	10	6	11	25	52	2.1

Subtotal	120	273	314	1,832	2,539	100

**Table 4:** Phlebotomine sandflies captured with HP trap in Governador Valadares by district and sex (from May 2011 to January 2012).

Year	Months	Districts																*N*	%
		Altinópolis		Mãe de		N. Sra.		Santa		Ilha dos		Santos		São		Turmalina			
				Deus		Graças		Helena		Araújos		Dumont		Raimundo					
		♀	♂	♀	♂	♀	♂	♀	♂	♀	♂	♀	♂	♀	♂	♀	♂		
2011	May	17	28	58	278	8	27	32	98	0	0	0	1	0	0	0	1	548	21.6
	Jul	8	34	20	153	7	49	19	72	0	0	0	0	0	2	0	0	364	14.4
	Sep	3	5	4	8	5	10	7	29	0	0	1	5	0	0	0	0	77	3.0
	Nov	21	33	15	7	8	25	11	57	3	1	0	0	0	1	3	4	189	7.4

2012	Jan	19	299	99	315	56	549	10	14	0	0	0	0	0	0	0	0	1,361	53.6

Total		467		957		744		349		4		7		3		8		2,539	100
